# Metamaterial-Inspired Flat Beamsteering Antenna for 5G Base Stations at 3.6 GHz

**DOI:** 10.3390/s21238116

**Published:** 2021-12-04

**Authors:** João Ricardo Reis, Mário Vala, Tiago Emanuel Oliveira, Telmo Rui Fernandes, Rafael Ferreira Silva Caldeirinha

**Affiliations:** 1 Polytechnic of Leiria, 2411-901 Leiria, Portugal; tiago.s.oliveira@ipleiria.pt (T.E.O.); telmo.fernandes@ipleiria.pt (T.R.F.); rafael.caldeirinha@ipleiria.pt (R.F.S.C.); 2 Instituto de Telecomunicações, 2411-901 Leiria, Portugal; mario.vala@co.it.pt; 3 Instituto de Superior Técnico, 1049-001 Lisbon, Portugal

**Keywords:** 5G, antenna, base-station, beamsteering, metamaterials, metasurface

## Abstract

In this paper, a metamaterial-inspired flat beamsteering antenna for 5G applications is presented. The antenna, designed to operate in the 3.6 GHz at 5G frequency bands, presents an unique flat form factor which allows easy deployment and low visual impact in 5G dense scenarios. The antenna presents a multi-layer structure where a metamaterial inspired transmitarray enables the two-dimensional (2D) beamsteering, and an array of microstrip patch antennas is used as RF source. The use of metamaterials in antenna beamsteering allows the reduction of costly and complex phase-shifter networks by using discrete capacitor diodes to control the transmission phase-shifting and subsequently, the direction of the steering. According to simulations, the proposed antenna presents steering range up to $\pm 20^{\circ }$, achievable in both elevation and azimuth planes, independently. To prove the concept, a prototype of the antenna has been built and experimentally characterised inside an anechoic chamber. Although constructed in a different substrate (FR4 substrate) as initially designed, beamsteering ranges up to $8^{\circ }$ in azimuth and $13^{\circ }$ in elevation, limited to the proposed case-studies, are reported with the prototype, validating the antenna and the usefulness of the proposed design.

## 1. Introduction

The 5th generation of mobile network (5G) has been the focus of research in the past few years. The main concept of 5G is to provide a highly flexible and scalable network technology for connecting everyone and everything, everywhere [1]. To date, many 5G systems are already being deployed worldwide, at the same time that the 3GPP 5G Release-18 is kicking in [2].

To comply with requirements of Enhanced Mobile Broadband (eMBB), Ultra Reliable Low Latency Communications (URLLC) and Massive Machine Type Communications (mMTC) (5G use cases [1]), and to cope with the associated growth of users and devices, the reduction of the coverage area (cell size) and the implementation of pico-cells is a trend in 5G [3]. However, the major issue associated with the reduction of covering areas is the consequent increase of cell number (to cover the same area) and thus, the excessive physical deployment of base station (or access point) antennas [3], causing a huge visual impact [4] particularly in dense urban locations. This leads to a high demand for hidden/concealed antennas with enclosures that allow for the reduction of the visual impact of such massive antenna deployment, e.g., antennas embedded in lump poles, fake trees or masked in building facades.

In an antenna engineering perspective, two major fronts are being tackled when designing 5G antennas: (i) antenna for (mobile) user equipment, which aim at the design of miniaturised antennae [5,6], whilst enabling multiple frequencies of operation [7,8,9], Multiple-Input Multiple-Output (MIMO) for enhanced signal processing and beamsteering, refs. [8,10,11]; (ii) the design of novel smart base station/access point antennas [12,13,14] that should enable, besides massive MIMO, beamsteering to aim at specific directions in time and space, while keeping moderate sizes and appellative shapes.

In particular, beamsteering is a desired technique in any wireless communication system leading to increased signal-to-noise ratio by redirecting the main beam of an antenna radiation pattern towards the receiver [15]. The most common solution to achieve beamsteering is using an antenna array coupled to a phase shifting mechanism [15,16]. Although widely used, such systems tend to be costly and complex specially for large arrays, due to necessity of a phase shifting device per array-element, to perform 2D beamsteering. Alternatively, transmitarray are being presented as a feasible alternative to such traditional beamsteering systems.

**Figure 1:**
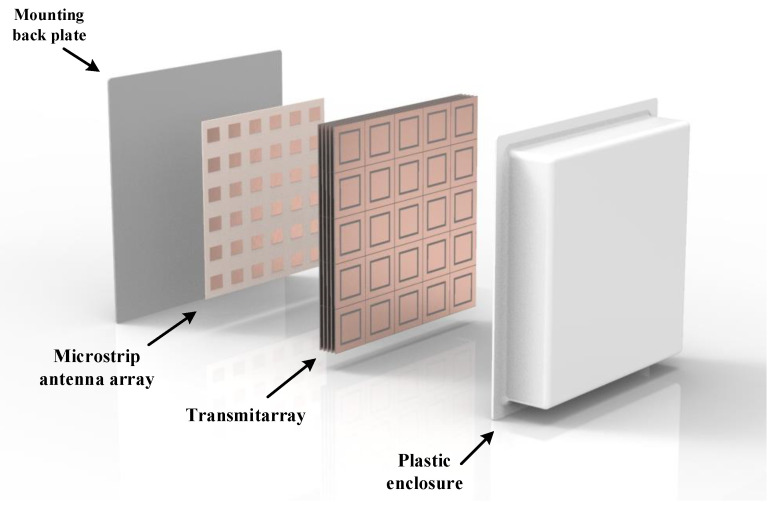
Overall schematic of the proposed 5G antenna design.

Transmitarrays are typically placed over a directional antenna aperture, as illustrated in Figure 1, with the aim of altering its radiation properties and, e.g., to perform focus, beamsteering or beam-forming. Following a similar physical principle as in the phased array [15], transmitarray are often comprised of microstrip patches, Frequency Selective Surfaces (FSS) and/or metamaterial unit-cells, assisted by the use of discrete components such p-i-n [17,18] or varactor diodes [19,20], to help tuning both frequency and phase response (phase-shift), on-demand.

In particular, the authors in [17] have designed a FSS-based unit-cell loaded with p-i-n diodes. When integrated in a 12×12 transmitarray, electronic beamsteering up to $\pm 40^{\circ }$, in both azimuth and elevation planes, can be achieved at 11.5 GHz. Similarly, in [18], a transmitarray using p-i-n diodes is also presented. The work remarkably reports $\pm 50^{\circ }$ of two-dimensional beamsteering at 12.5 GHz. Alternatively in [19], a FSS-inspired transmitarray is presented. This reconfigurable transmitarray enables 2D electronic beamsteering by using varactor diodes. Electronic beamsteering up to ±28${}^{\circ }$, in the main antenna planes, are achieved. Later in [20], the authors present a 6×6 transmitarray with a microstrip patch based unit-cell, loaded with varactor diodes, to electronically perform beamsteering up to $\pm 50^{\circ }$, at 24.6 GHz. Other transmitarray implementations can be found in the review paper [21]. It should be noted though that albeit the work presented in [17,18,20] reports slightly large beamsteering ranges, the final antenna assemblies are also bulky, as the transmitarrays are distanced enough from the source antenna so they can be placed in its focal point. This concept differs from the one presented in [19], where the transmitarray is placed in the aperture of the radiation source acting as a filtering antenna.

In this paper, a metamaterial-inspired flat beamsteering antenna for 5G base station operating in the 3.6 GHz frequency band, is presented. This follows the work initially presented by the authors in [22]. The proposed antenna, comprises a stacked layer design including a microstrip patch antenna array for feeding and, a transmitarray structure for beamsteering. The transmitarray, seen as the core of this work, is composed of square-slot resonating unit-cells loaded with capacitor diodes, capable of performing beamsteering in the two main antenna planes, as already presented in [19,23,24]. This works differs from the most presented in the literature, including [19,23,24], by using a microstrip patch array as feeding source replacing the typical feeding horn antennas, reducing the overall thickness of the apparatus.

Due to its the compact and light-weight format, the proposed antenna design is sought as a possible solution to a base station antenna when applied in a ceiling or in a building facade without visually compromising the surroundings. Therefore, building upon the work presented in [22], this paper details the work on optimisation, implementation and experimental characterisation of a prototype of the flat beamsteering antenna, which has been produced and characterised in terms radiation properties inside an anechoic chamber. Beamsteering ranges up to $8^{\circ }$ in azimuth and $13^{\circ }$ in elevation, limited to the proposed case-studies, are reported with the prototype, validating the antenna and the usefulness of the proposed design.

This paper is organised as follows: Section 2 presents the physical layout of the proposed antenna, while Section 2.2 introduces the mode of operation of proposed antenna while specifying the details to enable beamsteering. Subsequently, in Section 2.3, the simulation results obtained on the transmitarray antenna are being presented, followed by a critical discussion. Finally in Section 6, conclusions of the developed work are presented.

## 2. Antenna Design: Proof of Concept

### 2.1. Proposed Antenna

The antenna design being proposed in this paper is depicted in Figure 1. The antenna is composed of three main parts: (i) a microstrip patch antenna array operating as a feeding source; (ii) a transmitarray structure to control the wave front direction and thus, enable beamsteering and, finally (iii) a plastic enclosure to cover all electronic parts and provide a clean design and application. Each part is thoroughly detailed in this section.

The microstrip array, composed of a 6×6 elements, has been designed following the recommendations of [15,16] and assisted by the array design tool provided by CST Microwave Studio (MWS). A probe fed microstrip patch antenna has been used as unitary element of the array. After a complete set of simulation and subsequent optimisation in CST, the 6×6 array with patch dimensions of 23.9×19 mm${}^{2}$, separated by 41.6 mm (λ/2 at 3.6 GHz) in both horizontal and vertical direction, printed over a FR4 substrate ($\epsilon_{r}=4.4$ and tanδ = 0.025), presents a resonant frequency centred at 3.6 GHz, a bandwidth of 130 MHz and total gain of 18 dBi. For the sake of demonstration, an ideal feeding network has been considered, by forcing a simultaneous multi-port excitation with the same amplitude and phase in each array element, in the simulation environment.

**Figure 2:**
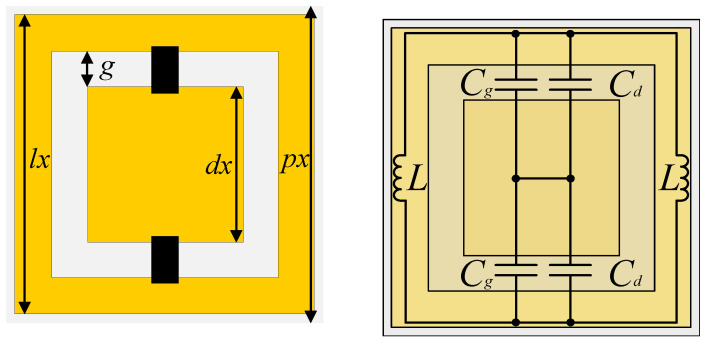
(**a**) Stacked unit-cell and (**b**) equivalent circuit.

The transmitarray, already presented by the authors in [19,23,24] at various frequencies, has been re-designed and optimised to operate at 3.6 GHz. The transmitarray follows a stacked layer design of square-slot resonating unit-cells loaded with capacitor diodes, as depicted in Figure 2. The square-slot cell exhibits a band-pass filtering characteristic operating as a frequency selective surface (FSS), which both central frequency and transmission phase-shift are controlled, on-the-fly, by the modifying the value of discrete capacitors placed between the inner patch and outer ring (Figure 2). Thus, any vertically polarised (TE) incident wave that impinges the transmitarray structure, is re-transmitted with a specific direction defined by the amount of phase delay introduced by each transmitarray element, operating very similar to planar antenna phased array [15].

**Figure 3:**
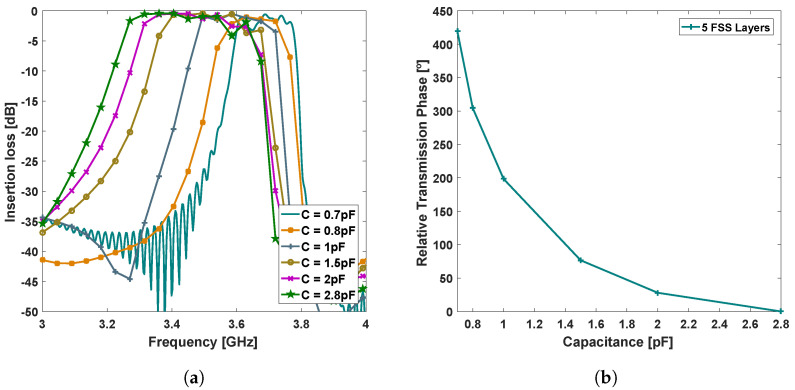
(**a**) Simulated S_21_ and (**b**) relative transmission phase depending on inserted capacitance.

In particular, a transmitarray element composed of 5 stacked layers of the unit-cell represented in Figure 2, with dimensions dx=36 mm, lx=59 mm, px=60 mm, gx=3 mm, etched on a Nelco NX9250 substrate ($\epsilon_{r}=2.5,tan\delta =0.0017$) with 1.57 mm of thickness, presents the filtering and transmission phase response illustrated in Figure 3, when the capacitance range is varied from 0.7 to 2.8 pF. The stacking of FSS layers, herein at 5 layers separated by 5mm, is a well-known method to increase the overall transmission phase shifting (and the order of the spatial filter), as reported in the literature [25], and used herein to enable antenna beamsteering. The principle of two-dimensional beamsteering with a transmitarray is thoroughly described in Section 2.2.

According to simulations, the proposed transmitarray element configuration exhibits an effective bandwidth of 100 MHz centred at 3.6 GHz (Figure 3a), defined by the maximum and minimum cut-off frequency of the lower and higher capacitor values, respectively. The global insertion loss is better than 4 dB, for every filtering configuration within the unit-cell bandwidth. Moreover, the relative transmission phase-shift is of $420^{\circ }$, within the capacitance sweep range (defined from 0.7 to 2.8 pF), respecting therefore the minimum requirement of $360^{\circ }$ to obtain full control of the beamsteering, as reported in [23].

In the final antenna configuration, the transmitarray and the microstrip array are separated by 20 mm and, protected with by a 3mm thick low-loss plastic enclosure, which confers to the proposed design an overall dimension of 350×350×60 mm${}^{2}$. While the spacers and the enclosure material have not been considered in the presented simulations, in a practical implementation these can be implemented with low-loss dielectric material, such as PTFE ($\epsilon_{r}=2.1,tan\delta =0.0002$), at the expense of minimally increasing the excess loss.

### 2.2. Enabling 2D Beamsteering with a Transmitarray

The method of implementing beamsteering with a transmitarray is well detailed in the literature, and particularly in the authors past publication [19,23]. In fact, it is built on the theory of planar antenna arrays [15,16], where a progressive phase-shift between adjacent elements should occur along the *X* and *Y* directions of a M×N array so 2D beamsteering could be enabled. Thus, when an incident EM wave impinges a M×N transmitarray structure, it suffers a local phase-delay $\alpha_{m,n}$ in every array element, causing the resultant (re-transmitted) wave to be steered in direction.

As demonstrated in [23], the relation between the two dimensional output directions (Azimuth and Elevation) of the steering angle, and the progressive phase-delay in the transmitarray, is given by (1),

(1) $$\left\{\begin{array}{c} \psi_{x}=-k_{0}.p.cos(El).sin(Az)\\ \psi_{y}=-k_{0}.p.sin(El) \end{array}\right.,$$

where $\psi_{x}$ and $\psi_{y}$ are the progressive phase along *X* and *Y* axis, respectively, and *p* is the periodicity of the p×p array elements. This can be represented in a by a relative phase matrix distribution, as in (2),

(2) $$\begin{array}{cc} & ⟶\psi_{x}\\ P_{m,n}=\psi_{y}↓ & \left[\begin{array}{cccccc} \alpha_{1,1} & .. & .. & .. & .. & \alpha_{1,n}\\ .. & .. & .. & .. & .. & ..\\ .. & .. & .. & .. & .. & ..\\ \alpha_{m,1} & .. & .. & .. & .. & \alpha_{m,n} \end{array}\right] \end{array}$$

where $\alpha_{m,n}$ is the phase delay introduced by each individual (*m*,*n*) element of the M×N transmitarray.

(3) $$\begin{array}{cc} & ⟶\psi_{x}\\ C_{m,n}=\psi_{y}↓ & \left[\begin{array}{cccccc} C_{1,1} & .. & .. & .. & .. & C_{1,n}\\ .. & .. & .. & .. & .. & ..\\ .. & .. & .. & .. & .. & ..\\ C_{m,1} & .. & .. & .. & .. & C_{m,n} \end{array}\right] \end{array}\;$$

**Figure 4:**
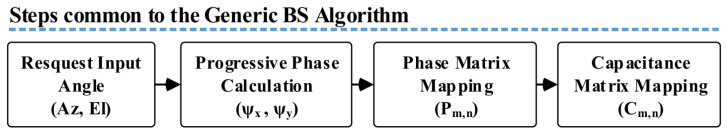
Simulated and measured $S_{2,1}$-parameter.

In a practical implementation with a transmitarray, such local phase-delay ($\alpha_{m,n}$) is, then given by unitary transmission phase, i.e., the phase difference between the incoming and outgoing wave in each array element. This is in turn function of the capacitors values loaded into each unit-cell, as demonstrated in Section 2. Therefore, for each beamsteering output angle, given by the pair (Az,El), will correspond a different capacitance matrix, herein represented by (3). For the sake of simplification, Figure 4 demonstrates the workflow for the computation of the capacitance matrices for a required output angle.

To assist with the computation, a Matlab script was developed to implement the beamsteering algorithm and estimate the capacitance values to apply in each unit-cell of the array for a desired output angle, with Az and El components (2D beamsteering). The script runs based on the Equations (1) to (3), and following the data flow of Figure 4.

The script starts by calculating the progressive phase ($\psi_{x}$, $\psi_{y}$) for the requested output angle pair (Az,El), in the X and Y direction of the array and, consequently, the theoretical phase-shift necessary to apply in each transmitarray element. Additionally, the (normalised) phase-shifts of every array element are mapped in a matrix ($P_{m,n}$), ranging from $0^{\circ }$ to $360^{\circ }$. The matrix has the size of the proposed structure (6 × 6 elements), wherein the position of first element of the matrix corresponds to the top left element of the transmitarray. Subsequently, the $C_{d}$ values of each element are extracted by linearly interpolating (mathematical operation) the correspondent transmission phase curve of Figure 3b with the phase-shifts given by the matrix $P_{m,n}$. Finally, the script returns a capacitance matrix $C_{m,n}$ indicating the capacitance values to apply in transmitarray element.

When no beamsteering is intended, i.e., the main lobe of the radiation pattern remains at broadside ($Az=0^{\circ }$ and $El=0^{\circ }$), all the capacitors of the transmitarray must be set to the same value, in order to cancel the progressive phase between elements, and therefore any steer in direction. For other angular output cases, specific pattern matrices are generated as it will be further indicated next section.

### 2.3. Initial Simulation Results

To validate the beamsteering capability, the antenna configuration depicted in Figure 1 was then simulated in CST MWS, for various output angles. As previously mentioned, the beam steered angles were obtained by specifically defining the capacitance value of each element of the 5×5 transmitarray, according to the phase pattern defined by capacitance matrix previously calculated. In particular, the antenna was set to steer main lobe of the radiation pattern in both azimuth and elevation planes, independently. Two-dimensional radiation patterns are presented to evaluate the antenna performance.

**Figure 5:**
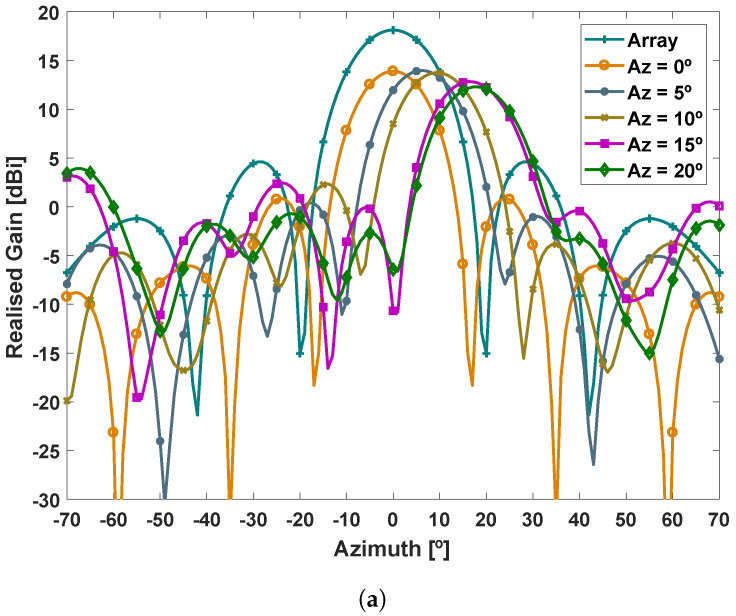

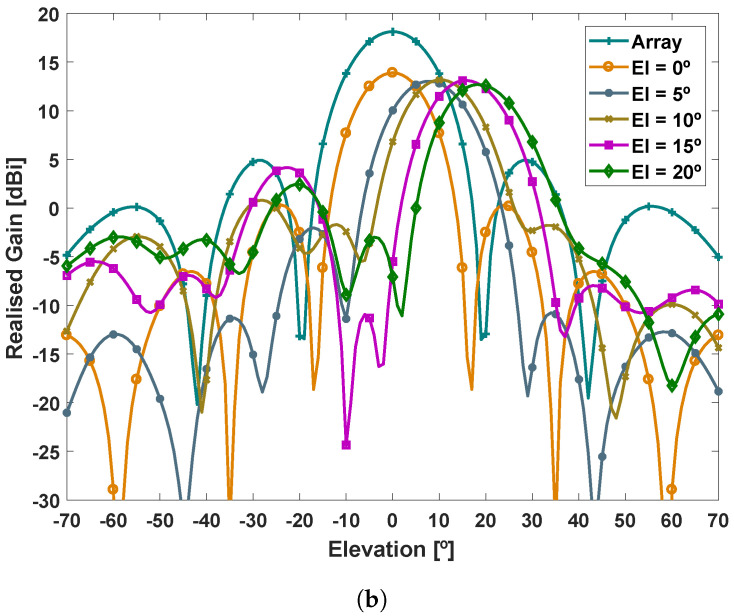
Simulated output angular sweep in the (**a**) azimuth and (**b**) elevation planes, respectively.

According to the simulations results of Figure 5, the antenna presents a maximum gain of 13.9 dBi when at boresight ($0^{\circ }$, $0^{\circ }$), against 18 dBi achieved with the microstrip array alone, which reflects the 4 dB of insertion loss caused by the transmitarray. Moreover, it is possible to observe that the antenna has the capability of performing beamsteering in a range defined $0^{\circ }$ and $20^{\circ }$, in both azimuth (Figure 5a) and elevation planes (Figure 5b), without major deformation of the original main lobe. Although simulations are only presented for an angular sweep in the positive part of the axis, the antenna presents a good symmetry around the Y-axis in both antenna planes, with a maximum achievable angle of $\pm 20^{\circ }$. Within the presented steering range, the maximum gain only decays of around 3 dB. For larger values, relatively high side lobes, with side lobe levels >7 dB start to appear.

## 3. Transmitarray Optimisation and Prototyping

**Figure 6:**
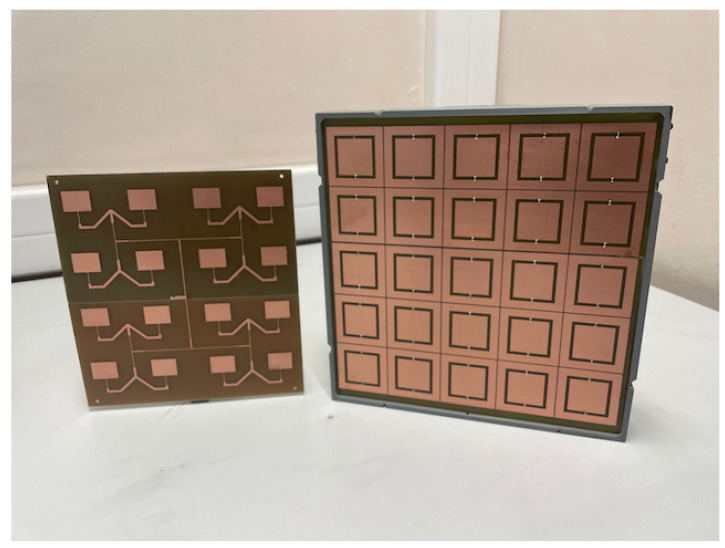
Microstrip feeding antenna (**left**) and transmitarray (**right**) prototypes.

To validate the beamsteering capabilities of the proposed antenna configuration, the microstrip antenna array and the 5×5 transmitarray have been prototyped, tested and experimentally characterised. Figure 6 depicts the prototype of both antenna and transmitarray models. In order to reduce the manufacturing time and the total implementation cost, both the transmitarray and the microstrip feeding antenna were produced in house, using the available FR4 substrate ($\epsilon_{r}=4.7$, tanδ=0.014 and substrate thickness 1.6 mm), instead of Nelco substrate considered in the initial simulations of Section 2. Such alteration came at the expense of reducing the overall antenna performance due to the poor EM properties (in particular, high loss tangent) of the FR4, specially at microwave frequencies.

This modification in substrate, led to the optimisation of the unit-cell dimensions and consequent adjustment in the capacitance range. Therefore, the optimised dimensions of the unit-cell using FR4 substrate are detailed in Table 1. This leads to a transmitarray total dimension of 300×300×30 mm${}^{3}$. To ensure the necessary separation distance between layers and avoid the tendency of the large layers of substrate to bend, a 3D printed substructure has been constructed. The structure, produced in PLA material using a standard 3D printing machine, allows to slide in each layer at the desired position, providing a easy method to remove a layer, when needed, e.g., to load a different capacitance pattern layer and thus obtain a different beamsteering output angle.

The microstrip feeding antenna has been designed with the aid of Antenna Magus, a software tool for antenna design and modelling process. In particular, the *"M-by-N rectangular patch array with corporate feed"* template has been used to generate the initial dimensions for a 4×4 microstrip array, which has been further optimised in CST MWS. Overall antenna dimensions are 230×230×1.6 mm${}^{3}$, comprising 16 microstrip patch elements with dimensions of 23.6×17.9 mm${}^{2}$, distanced by 56.7 mm in both array directions. The feeding antenna is also mounted to the transmitarray, using the mentioned 3D printed structure, separated 2 cm from the subsequent transmitarray layer.

In this particular demonstration, three case studies have been analysed, corresponding to three different capacitance patterns applied throughout the transmitarray:

− Case (i), when all capacitors are set to 1.2 pF, to evaluate the total insertion loss caused by the structures, causing the output angle to remain at boresight (Az,El) = ($0^{\circ }$,$0^{\circ }$);
− Case (ii), when the transmitarray is configured to steer the main lobe of the radiation pattern to the direction ($-10^{\circ }$,$0^{\circ }$), by loading the capacitance matrix (5), and finally,
  (4) $$|\psi_{x,y}|=\left[\begin{array}{ccccc} 154.2 & 115.6 & 77.1 & 38.5 & 0\\ 154.2 & 115.6 & 77.1 & 38.5 & 0\\ 154.2 & 115.6 & 77.1 & 38.5 & 0\\ 154.2 & 115.6 & 77.1 & 38.5 & 0\\ 154.2 & 115.6 & 77.1 & 38.5 & 0 \end{array}\right]\;{[}^{\circ }]$$
  (5) $$C_{x,y}=\left[\begin{array}{ccccc} 0.82 & 0.75 & 0.69 & 0.65 & 0.60\\ 0.82 & 0.75 & 0.69 & 0.65 & 0.60\\ 0.82 & 0.75 & 0.69 & 0.65 & 0.60\\ 0.82 & 0.75 & 0.69 & 0.65 & 0.60\\ 0.82 & 0.75 & 0.69 & 0.65 & 0.60 \end{array}\right]\;\left[\mathrm{pF}\right]$$
− Case (iii), when the transmitarray is set to steer the main lobe of the radiation pattern to the direction ($0^{\circ }$,$+15^{\circ }$), by loading the capacitance matrix (7).
  (6) $$|\psi_{x,y}|=\left[\begin{array}{ccccc} 229.8 & 229.8 & 229.8 & 229.8 & 229.8\\ 172.3 & 172.3 & 172.3 & 172.3 & 172.3\\ 114.9 & 114.9 & 114.9 & 114.9 & 114.9\\ 57.4 & 57.4 & 57.4 & 57.4 & 57.4\\ 0 & 0 & 0 & 0 & 0 \end{array}\right]\;{[}^{\circ }]$$
  (7) $$C_{x,y}=\left[\begin{array}{ccccc} 1.15 & 1.15 & 1.15 & 1.15 & 1.15\\ 0.88 & 0.88 & 0.88 & 0.88 & 0.88\\ 0.75 & 0.75 & 0.75 & 0.75 & 0.75\\ 0.67 & 0.67 & 0.67 & 0.67 & 0.67\\ 0.60 & 0.60 & 0.60 & 0.60 & 0.60 \end{array}\right]\;\left[\mathrm{pF}\right]$$

## 4. Experimental Setup

In order to validate the initial theoretical concept as well as the simulation results, the prototype of Figure 6 has been built and evaluated in terms of antenna matching and beamsteering characteristics. This has been performed through the analysis of $S_{1,1}$-parameter and 3D radiation patterns, respectively. All measurements were obtained from 3 to 4 GHz using a *R&S ZVM* vector network analyser (VNA).

**Figure 7:**
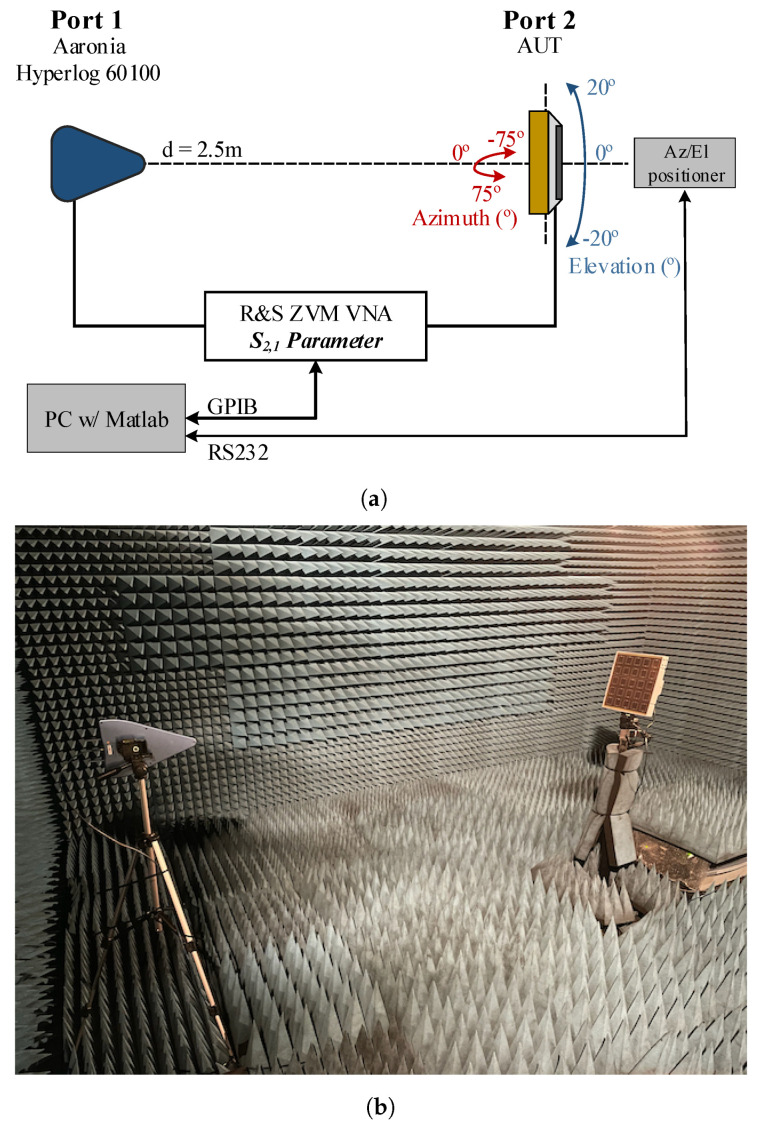
Experimental setup: (**a**) block diagram with side-view representation and (**b**) photography of the setup inside the anechoic chamber.

Particularly, the radiation patterns were obtained with the acquisition of the $S_{2,1}$-parameter, per angular step, using the setup depicted in Figure 7. At the transmitter end, a well characterised *Aaronia Hyperlog 60100* antenna connected to Port1 of the VNA and kept fixed throughout the measurements, was used. At the receiver end, a well characterised *Aaronia Hyperlog 30100* antenna, connected to Port 2 was used as reference (latter replaced by the antenna under test (AUT)). Both antennas were located 2.5 m apart to ensure that the measurements are performed in the far-field region.

In order to obtain the 3D radiation pattern, the transmitter antenna was kept fixed throughout the measurements, while receiver was rotated around its own axis, with the assist of motorised pan/tilt head unit. At each angular step within the range of $-75^{\circ }\leq Az\leq 75^{\circ }$ and $-20^{\circ }\leq El\leq 20^{\circ }$, the $S_{2,1}$-parameter was acquired and referenced to the one when using the reference antenna, following the gain transfer method procedure described in [26]. Prior to a measurement, antennas were aligned to the maximum radiated power direction. The synchronization between the $S_{2,1}$ data acquisition and movement control, was executed in Matlab using an in-house developed software routine. To avoid any external electromagnetic contamination and to obtain precise and clean measurement results, all measurements were obtained inside an anechoic chamber (Figure 7b).

## 5. Results and Discussion

**Figure 8:**
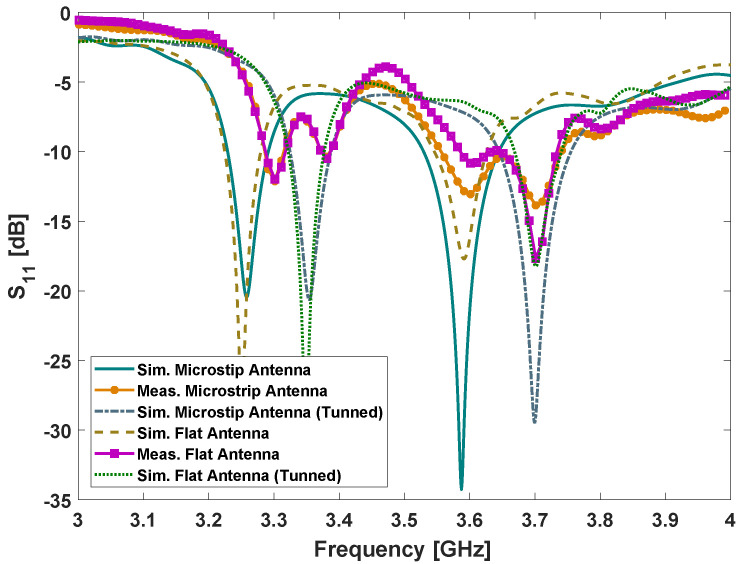
Simulated and measured $S_{1,1}$-parameter.

To understand the initial behaviour of the proposed antenna configuration, the $S_{1,1}$ parameters are presented and compared in Figure 8, for both simulations and measurements, with and without the transmitarray attached to the feeding antenna. As it can be observed, simulated results for the microstrip array indicate that the antenna is resonating at 3.58 GHz (considering the $S_{1,1}<$−10 dB criteria), presenting a bandwidth of 110 MHz. Despite the fact another resonance frequency is present around 3.25 GHz, this frequency point is not considered in the analysis, as it falls outside the proposed transmitarray operating range.

When the transmitarray, with all the capacitors set at 1.2 pF (case study (i)), is placed in the vicinity of the feeding antenna, the $S_{1,1}$ remains almost unaltered, despite a slight reduction in the bandwidth to 80 MHz, according to simulations. However, when comparing the simulated results with the ones obtained from experiments, an up-shift of 105 MHz in the entire $S_{1,1}$ curves is noticed and, a resonating peak can be observed at 3.7 GHz, with a bandwidth of approximately 75 MHz, corresponding the effective bandwidth of the transmitarray. This offset between experimental and simulation result has been further studied and found to be associated with substrate permittivity of the FR4, which has been considered to be 4.7 in the antenna/transmitarray design stage, respecting the available manufacturer data ($\epsilon_{r}=4.7\;\;@$1 MHz [27]). After a parametric study in CST MWS, an $\epsilon_{r}=4.4$ was found to provide a better match between the simulated and experimental $S_{1,1}$-parameter, as depicted in Figure 8. As this finding was only noticed after the analysis of the measurements results, and for the sake of the evaluation, beamsteering capabilities of the flat antenna design are therefore characterised at 3.6 GHz for simulations, but at 3.7 GHz for experiments.

**Figure 9:**
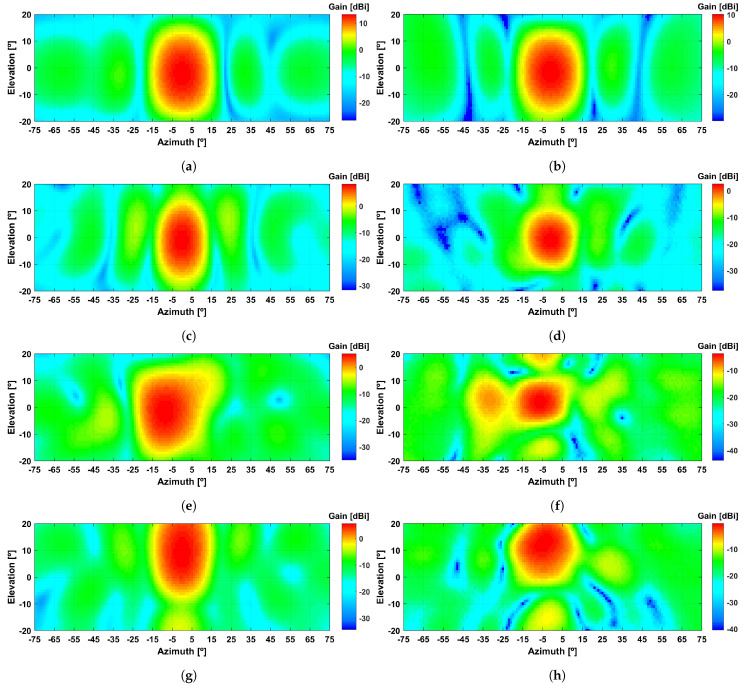
Simulated and measured 3D radiation patterns for: (**a**,**b**) microstrip array and; transmitarray set to steer towards: (**c**,**d**) $Az=0^{\circ }$ and $El=0^{\circ }$, (**e**,**f**) $Az=-10^{\circ }$ and $El=0^{\circ }$ and (**g**,**h**) $Az=0^{\circ }$ and $El=15^{\circ }$.

In terms of beamsteering characteristics, the radiation patterns of Figure 9, summarised in Table 2, which depict the simulated and experimental results side-by-side, show a relatively good agreement between both sets of results. As a global appreciation, it is possible to observe a clear similarity in radiation patterns shape, for all the considered case-studies (detailed in Section 3). In particular, when analysing the results for the reference microstrip feeding array, the antenna presents 13.2 dBi of realised gain in simulations (Figure 9a), against 10.2 dBi in experiments (Figure 9b). Moreover, it presents an half-power beam width (HPBW) of 19${}^{\circ }$ in both elevation and azimuth planes, according to simulations, and of 18${}^{\circ }$ and 19${}^{\circ }$, according to measurements, in the same planes, respectively.

Furthermore, when analysing the results of case study *i)*, where all capacitors within the transmitarray are set to 1.2 pF (Figure 9c,d, the inclusion of the transmitarray introduces a minor offset in the main lobe direction (−2${}^{\circ }$ in simulation and −1${}^{\circ }$ in experiments), sought to be associated with the vertical displacement of the feeding antenna, in relation to the centre of transmitarray. At the maximum gain direction, a realised gain of 8.2 and 2.6 dBi is obtained in simulation and experiments, respectively. When comparing such results to the ones for the microstrip feeing antenna isolated, it is possible to estimate the insertion loss of the transmitarray in 5.2 dB, according to simulations and, 7.7 dB according to experiments. Although relatively high insertion losses are noticed, especially for the prototype, better results could have been obtained with a proper RF substrate as indicated in the initial transmitarray design presented in Section 2.

In terms of beamsteering capabilities, the results from Figure 9e–h, indicate that it is possible to steer the direction of the main lobe of the reference radiation pattern, therefore validating the proposed antenna design. As for case study (ii) (Figure 9e,f, where the main lobe is set to steer towards ($-10^{\circ }$,$0^{\circ }$), it can be noticed that the main power direction is set at ($-9^{\circ }$,$-2^{\circ }$) with a gain of 5.2 dBi, in simulations. On the other hand, experimental results report the main power direction set at ($-8^{\circ }$,$-1^{\circ }$), with a maximum gain of −0.6 dBi, only $2^{\circ }$ short from the expected angle. For case study (iii), simulation results report the main lobe of the radiation pattern set at ($0^{\circ }$,$12^{\circ }$), resulting in a effective steering range of $14^{\circ }$, due to the initial offset of $-2^{\circ }$ in the elevation plane, noticed in case study (ii). This is remarkably close to the results obtained in experiments, where an effective steering range of $14^{\circ }$ is also noticed. Such discrepancies between simulated and experimental output beamsteering angles are sought to be related with round off errors between theoretical values of relative phase/capacitance, and the surface mount capacitors used for prototyping. Nevertheless, the accuracy of the output angles is acceptable specially when compared to the large HPBW of the main lobe of the radiation pattern. Moreover, if applied to an automated model where capacitance could be electronically controlled, e.g., using varactor diodes, the angular error could compensated electronically.

Even though the realised gain is relatively low in experiments, due to the insertion losses of the transmitarray, it presents a similar difference in magnitude for both case-studies (ii) and (iii), with a relation of proximately 3 dB decrease in simulated gain, and 2 dB in experiments, when steering from broadside, to ($-10^{\circ }$,$0^{\circ }$) and/or ($0^{\circ }$,$15^{\circ }$).

## 6. Conclusions

This paper presents a flat beamsteering antenna for 5G applications in the 3.6 GHz frequency bands. The proposed antenna presents a multi-layer structure composed of a microstrip patch array and a metamaterial inspired transmitarray structure that enables 2D-dimensional beamsteering, which replace the costly and complex phase-shifter networks. The proposed antenna presents in simulations 13.9 dBi of gain, 100 MHz of effective bandwidth with a maximum beamsteering range defined between $\pm 20^{\circ }$, achievable in both elevation and azimuth planes, independently. A prototype of the antenna has been built and experimentally characterised inside an anechoic chamber. Although constructed in a different substrate (FR4 substrate), to reduce implementation cost and construction time, from the initial design, relatively good results in terms of beamsteering are achieved. Beamsteering ranges up to $8^{\circ }$ in azimuth and $13^{\circ }$ in elevation, limited to the proposed case-studies, are reported with the prototype, therefore validating the proposed antenna design. Besides its relatively good performance, which can be further improved with a careful selection of the RF substrates, the proposed antenna also presents an unique compact and flat form factor with a moderate size and appellative shapes, which allows an easy deployment whilst reducing the visual impact in 5G dense scenarios, when comparing with traditional base-station antenna.

## Figures and Tables

**Table 1:** Design parameters for transmitarray operating at 3.6 GHz.

Unit-Cell Dimensions		FR4 Substrate	
Parameter	Value (mm)	Parameter	Value
dx	36		
lx	50.8	ϵ _r_	4.7
px	51.8	tan(δ)	0.014
gx	3	thickness	1.6 mm
layer separation	5		

**Table 2:** Summary table for simulated and measured radiation pattern.

		Simulation @3.6 GHz			Experiments @3.7 GHz		
Expected		Main Lobe			Main Lobe		
**Az** ** $\left({}^{\circ }\right)$ **	**El** ** $\left({}^{\circ }\right)$ **	**Az** $\left({}^{\circ }\right)$	**El** $\left({}^{\circ }\right)$	**Gain [dBi]**	**Az** $\left({}^{\circ }\right)$	**El** $\left({}^{\circ }\right)$	**Gain [dBi]**
*-	*-	0	0	13.2	0	0	10.2
0	0	0	−2	8.2	−1	−1	2.6
−10	0	−9	−2	5.2	−8	−1	−0.6
0	15	0	12	5.4	−2	13	−0.3

* Microstrip array without the transmitarray attached.
